# Integrated Pan-Cancer, Single-Cell, and Spatial Transcriptomic Analyses Identify ZDHHC12 as a Biomarker Associated with Macrophage Infiltration and the Immune Landscape in Glioma

**DOI:** 10.7150/jca.136931

**Published:** 2026-09-03

**Authors:** Chao Zhang, Da Teng, Yu Wang, Shiqiang Hou, Wenjun Zhang, Ning Lin

**Affiliations:** 1 Department of Neurosurgery, The Affiliated Chuzhou Hospital of Anhui Medical University, The First People's Hospital of Chuzhou, Chuzhou 239000, China.; 2 Department of Hepatobiliary Surgery, The First Affiliated Hospital of Anhui Medical University, Hefei 230001, China.; 3 Department of Hepatobiliary Pancreatic and Splenic Surgery Ward I, The Affiliated Chuzhou Hospital of Anhui Medical University, The First People's Hospital of Chuzhou, Chuzhou 239000, China.

**Keywords:** ZDHHC12, GBM, tumor microenvironment, tumor-associated macrophages, pan-cancer

## Abstract

**Background:**

The tumor immune microenvironment (TME) critically influences cancer progression and therapeutic response. However, the pan-cancer expression landscape, prognostic relevance, and spatial distribution of ZDHHC12 remain incompletely characterized. This study investigated the prognostic value of ZDHHC12 and its associations with immune microenvironmental features and drug sensitivity.

**Methods:**

Data from The Cancer Genome Atlas (TCGA) and the Genotype-Tissue Expression (GTEx) datasets were used to evaluate ZDHHC12 expression and prognosis across cancer types. Immune infiltration analyses, single-cell RNA sequencing, and spatial transcriptomics were integrated to characterize the associations of ZDHHC12 with the cancer immunity cycle and the spatial architecture of glioma. Drug sensitivity and immunotherapy-related metrics were assessed using pharmacogenomic databases and computational prediction models.

**Results:**

ZDHHC12 was aberrantly expressed across multiple tumors and was associated with patient prognosis. Its expression was broadly correlated with immune cell recruitment- and activation-related signatures. In glioma, single-cell and spatial transcriptomic analyses showed enrichment of ZDHHC12 in monocyte/macrophage populations and spatial co-localization with BAK1, CD68, and CD163. ZDHHC12 expression was also associated with predicted drug sensitivity and immunotherapy-related metrics.

**Conclusion:**

ZDHHC12 may serve as a candidate pan-cancer prognostic biomarker. In glioma, its expression is associated with macrophage-enriched and immunosuppressive microenvironmental features. Functional studies are required to establish causality and determine its therapeutic relevance.

## 1. Introduction

Malignant tumors remain one of the leading causes of morbidity and mortality worldwide, posing a serious threat to human health. With the rapid advancement of high-throughput multi-omics technologies, pan-cancer analyses have provided an unprecedented macroscopic perspective for elucidating shared oncogenic mechanisms across different cancer types, identifying co-expression modules, and dissecting the heterogeneity of the tumor microenvironment (TME)^1^. Within the highly complex TME, the extent of immune cell infiltration, spatial distribution, and polarization status not only profoundly influence tumor proliferation, invasion, and immune evasion but also largely determine patient responses to novel immunotherapies, such as immune checkpoint inhibitors^2^,^3^. Consequently, the systematic identification of novel biomarkers capable of characterizing the immune landscape of the TME, accurately predicting patient prognosis, and assessing drug sensitivity has emerged as a critical scientific priority in the field of precision oncology.

Among the vast spectrum of malignant tumors, glioma—the most common and aggressive primary malignancy of the central nervous system—poses substantial therapeutic challenges^4^. Due to its profound cellular heterogeneity and an extremely immunosuppressive microenvironment, the efficacy of conventional radiotherapy, chemotherapy, and immunotherapy remains markedly limited in glioma^3^. The glioma microenvironment is densely populated by tumor-associated macrophages (TAMs)^2^,^5^. Accumulating evidence indicates that the polarization of TAMs toward the M2 phenotype is closely linked to malignant progression and therapeutic resistance in glioma^3^,^6^. In recent years, breakthroughs in single-cell transcriptome sequencing and spatial transcriptomics have provided powerful technological platforms for dissecting the heterogeneity of glioma cell populations and the complex spatial interaction networks of TAMs with high resolution across both temporal and spatial dimensions^7^.

Protein palmitoylation is a reversible lipid modification that occurs on a wide range of oncogenic and tumor suppressor proteins^8^. This modification is dynamically regulated by zDHHC palmitoyltransferases and protein depalmitoylases^9^, and is extensively involved in modulating the membrane localization, stability, and functional activity of numerous oncoproteins^10^. It is well established that protein palmitoylation participates in various key cellular processes by altering protein conformation, localization, stability, and function^11^,^12^. Emerging evidence has revealed that multiple ZDHHC enzymes and their mediated palmitoylation events contribute to tumorigenesis and influence therapeutic responses^13^-^15^. Recent studies have shown that ZDHHC15 is associated with glioma malignancy and can promote glioblastoma stem cell tumorigenicity through c-MET S-palmitoylation^16^,^17^. As a critical member of this family, ZDHHC12 has been shown to affect the sensitivity of ovarian cancer to targeted agents by mediating the palmitoylation of poly(ADP-ribose) polymerase 1 (PARP1) and is also involved in negatively regulating NLR family pyrin domain containing 3 (NLRP3) inflammasome assembly^18^,^19^. Nevertheless, the expression landscape, prognostic value, and immunological relevance of ZDHHC12 at the pan-cancer level remain to be systematically elucidated. In particular, the potential links, underlying mechanisms, and coordinated regulatory networks between ZDHHC12 and immune microenvironment infiltration, as well as its association with BAK1 in glioma, remain to be explored.

To address these knowledge gaps, the present study adopted a multi-omics integration strategy following a “pan-cancer to focal cancer” framework. First, using large-scale public datasets, we systematically evaluated the expression landscape, prognostic relevance, and immune infiltration-related features associated with ZDHHC12 across multiple cancer types. Subsequently, focusing on glioma, we integrated single-cell and spatial transcriptomic data to characterize the cellular distribution and spatial associations of ZDHHC12 with the general macrophage marker CD68, the immunoregulatory macrophage-associated marker CD163, and the co-expressed molecule BAK1. Finally, we performed exploratory analyses of the associations between ZDHHC12 expression, predicted drug sensitivity, and immunotherapy-related metrics (Flow diagram). Collectively, this study provides an integrated characterization of the pan-cancer and spatial transcriptomic landscape of ZDHHC12 and generates hypotheses regarding its potential association with macrophage-enriched immune features in glioma.

## 2. Methods

### 2.1. Data acquisition and download

Pan-cancer transcriptomic data and corresponding clinicopathological information (including survival time) were downloaded from The Cancer Genome Atlas (TCGA) database^20^. To address the lack of normal control samples, transcriptomic data from healthy human tissues were additionally obtained from the Genotype-Tissue Expression (GTEx) project (https://www.gtexportal.org/home/index.html)^21^. Before data integration, TPM expression values from TCGA and GTEx were transformed as log2(TPM + 1). Batch effects associated with dataset origin were adjusted using the ComBat function implemented in the sva R package. Dataset source (TCGA or GTEx) was specified as the batch variable, and biological grouping information was included in the model matrix to preserve biologically relevant variation.

### 2.2. Spatial Transcriptomics Data Processing

In this study, spatial transcriptomic analysis of pan-cancer tissue sections was performed using the GuangRe Bio database (https://www.grswsci.top) and the Cottrazm algorithm. This algorithm integrates single-cell transcriptomic data, spatial transcriptomic data, and spatial spot location information to predict the cellular composition of each spatial spot through a deconvolution approach. By combining spatial transcriptomic data with H&E-stained images, the algorithm precisely delineates tumor boundaries connecting malignant and non-malignant regions. Furthermore, leveraging single-cell transcriptomic data, spatial transcriptomic data, and spatial spot positional information, a deconvolution algorithm was employed to predict the cellular composition of each spatial spot and reconstruct cell type-specific gene expression profiles at sub-spot resolution.

### 2.3. Gene Expression Analysis

To validate the expression of target genes at the protein level, immunohistochemical staining images of multiple tumor types and corresponding normal tissues were obtained from the Human Protein Atlas (HPA) database (https://www.proteinatlas.org/)^22^. In addition, raw single-cell data for glioma were downloaded from the Gene Expression Omnibus (GEO) database for subsequent dissection of the TME.

### 2.4. Pan-Cancer Expression Landscape and Prognostic Assessment

The Wilcoxon rank-sum test was employed to analyze differential ZDHHC12 expression between tumor and normal tissues in the combined TCGA and GTEx cohort. Receiver operating characteristic (ROC) curves and the area under the curve (AUC) were used to evaluate the diagnostic value of ZDHHC12 in distinguishing various cancer types from their corresponding normal tissues. To explore the relationship between ZDHHC12 and pan-cancer patient prognosis, overall survival (OS) data were extracted. Univariate Cox proportional hazards regression models and Kaplan-Meier survival curves with log-rank tests were systematically applied to assess the association between ZDHHC12 expression levels and pan-cancer patient outcomes.

### 2.5. Tumor Microenvironment and Immune Infiltration Characterization

The ESTIMATE algorithm was applied to calculate immune scores, stromal scores, and tumor purity for pan-cancer samples^23^. To precisely assess the infiltration levels of various immune cell subsets within the TME, the CIBERSORT tool, based on a deconvolution algorithm, was further employed to estimate the relative abundances of distinct tumor-infiltrating immune cells^24^. Subsequently, Spearman correlation analysis was performed to investigate the relationship between ZDHHC12 expression levels and the degree of immune cell infiltration.

### 2.6. Single-Cell RNA Sequencing Data Processing and Cell Cluster Annotation

To dissect the heterogeneous distribution of ZDHHC12 in the glioma microenvironment at single-cell resolution, standard dimensionality reduction and clustering analyses were performed using the Seurat R package^25^. Strict quality control was first applied to filter out low-quality cells. Subsequently, log-normalization was performed using the NormalizeData function, and highly variable genes were identified. Principal component analysis was conducted for linear dimensionality reduction, followed by batch effect correction across samples using the Harmony algorithm^26^. Nonlinear dimensionality reduction and cell population visualization were achieved using the UMAP algorithm.

### 2.7. Spatial Transcriptomics Analysis and Co-localization

Processing of glioma spatial transcriptomics data was performed using the spatial analysis module of the Seurat software. After filtering low-quality spatial spots and applying SCTransform normalization, H&E-stained tissue section images were precisely aligned with the spatial gene expression matrix. To investigate the spatial relationships among ZDHHC12, CD68, CD163, and BAK1, spatial feature distribution maps were generated for each of the four genes, followed by co-expression and co-localization scoring analyses of spatial feature domains. Furthermore, Pearson correlation analysis was performed to quantitatively validate the statistical association between ZDHHC12 and BAK1 expression levels within localized spatial regions.

### 2.8. Gene Set Enrichment Analysis (GSEA) and Correlation Analysis

To investigate the biological functions and signaling pathways associated with ZDHHC12 expression, patients within each cancer cohort were assigned to ZDHHC12-high and ZDHHC12-low groups according to the median ZDHHC12 expression value in the corresponding cohort. The cutoff was calculated separately for each cancer type. Gene set enrichment analysis was performed using the clusterProfiler R package^27^. KEGG and Hallmark gene sets were obtained from the Molecular Signatures Database. An adjusted P-value < 0.05 and a false discovery rate < 0.25 were considered statistically significant.

### 2.9. Biological Function Annotation

Functional annotation of differentially expressed genes was performed using the clusterProfiler R package to systematically evaluate their functional associations. For Gene Ontology (GO) terms and Kyoto Encyclopedia of Genes and Genomes (KEGG) pathways, categories with both P-value and q-value < 0.05 were considered statistically significant.

Functional states corresponding to distinct tumor cell types were characterized using the CancerSEA database^28^. Gene set variation analysis (GSVA) algorithm was applied to perform gene set variation analysis, generating a comprehensive score for each gene set to evaluate potential alterations in biological functions across samples^29^.

### 2.10. Immune Cell Infiltration Analysis

The association between ZDHHC12 expression levels and immune cell infiltration was assessed using the TIMER 2.0 database (http://timer.cistrome.org/)^30^. TCGA samples were stratified according to six predefined immune subtypes for subsequent analyses, including C1 (wound healing), C2 (IFN-γ dominant), C3 (inflammatory), C4 (lymphocyte depleted), C5 (immunologically quiet), and C6 (TGF-β dominant)^31^. Spearman correlation analysis was employed to calculate correlations between variables.

### 2.11. Drug Sensitivity Screening and Immunotherapy Response Prediction

To explore the associations between ZDHHC12 expression and predicted drug response in glioma, the pRRophetic package was employed to predict clinical chemotherapy responses based on gene expression data^32^. Drug sensitivity was further assessed using the CTRP 2.0 database (https://portals.broadinstitute.org/ctrp.v2.1/) and the PRISM drug repurposing dataset (https://depmap.org/portal/prism/) via the R package "oncoPredict"^33^. Drug sensitivity analysis was conducted to identify compounds whose response metrics were associated with ZDHHC12 expression, with the algorithm primarily constructing a ridge regression model based on the relationship between baseline gene expression levels and in vitro drug sensitivity in cell lines.

### 2.12. Statistical Analysis

All data processing and statistical analyses were performed using R software. Comparisons of continuous variables between two groups were conducted using the independent samples Wilcoxon rank-sum test, while comparisons among multiple groups were performed using the Kruskal-Wallis test. Correlations between variables were assessed using either Spearman's or Pearson's correlation coefficients, as appropriate. Survival analysis was carried out using the Kaplan-Meier method to generate survival curves, and the log-rank test was applied to evaluate the significance of survival differences between groups. In all statistical tests, a two-sided P-value < 0.05 was considered statistically significant.

## 3. Results

### 3.1. Pan-Cancer Expression Landscape, Diagnostic Value, and Prognostic Significance of ZDHHC12

**Figure 1:**
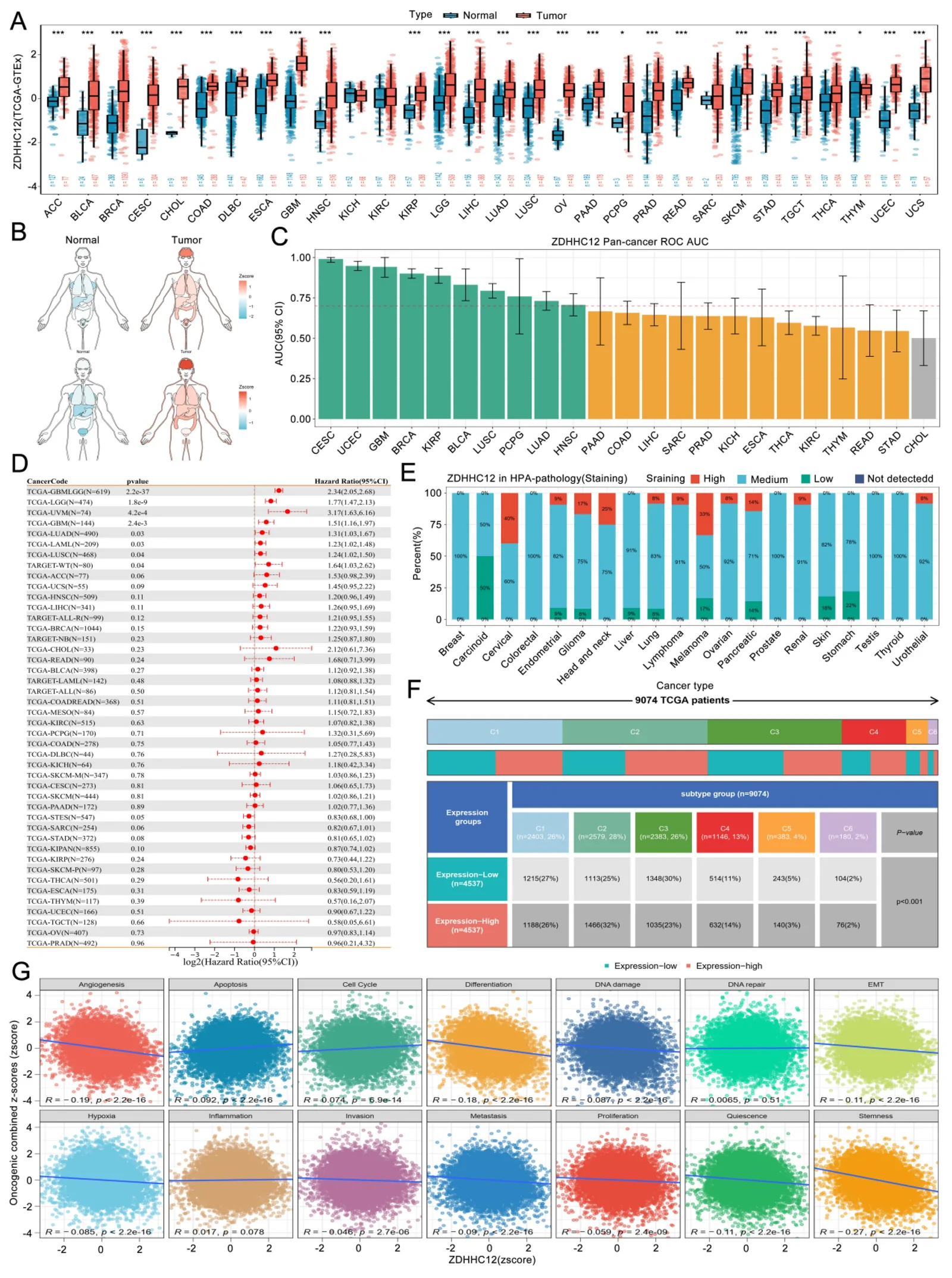
Pan-cancer expression landscape, diagnostic and prognostic value of ZDHHC12, and its correlation with oncogenic pathways. (A) Differential expression analysis of ZDHHC12 mRNA levels in solid tumor types and corresponding normal tissues based on the TCGA and GTEx databases. (B) Anatomical spatial expression distribution map of ZDHHC12 in human normal and tumor tissues. (C) Bar plot summarizing the area under the ROC curve (AUC) for evaluating the pan-cancer diagnostic efficacy of ZDHHC12. (D) Forest plot of univariate Cox regression analysis showing the prognostic value of ZDHHC12. (E) Stacked bar plot showing the distribution of pan-cancer IHC staining intensity. (F) Expression levels of ZDHHC12 in six immune subtypes. (G) Scatter plot illustrating the correlation between ZDHHC12 expression levels and enrichment scores (Z-scores) of 14 canonical oncogenic signaling pathways.

To systematically elucidate the potential role of ZDHHC12 in tumor initiation and progression, we first assessed its expression landscape at the pan-cancer level. Given the lack of sufficient normal control samples in certain TCGA tumor cohorts, we integrated data from TCGA and GTEx to construct a more comprehensive transcriptomic expression atlas^20^,^21^. Differential expression analysis revealed that ZDHHC12 mRNA levels were highly significantly aberrantly expressed in multiple malignancies compared with corresponding normal tissues (Fig. 1A). This cross-cancer aberrant expression pattern was visually corroborated at the anatomical level (Fig. 1B), suggesting that ZDHHC12 is broadly involved in the pathophysiological processes of various solid tumors.

We next evaluated the efficacy of ZDHHC12 as a broad-spectrum diagnostic biomarker using ROC curves. The results demonstrated that ZDHHC12 exhibited high sensitivity and specificity in distinguishing tumor from normal tissues. Quantitative analysis of the AUC showed that ZDHHC12 achieved notably high AUC values in multiple cancer types, including cervical squamous cell carcinoma and endocervical adenocarcinoma (CESC), uterine corpus endometrial carcinoma (UCEC), and glioblastoma (GBM) (Fig. 1C), highlighting its substantial potential as a pan-cancer diagnostic marker.

Having established the expression and diagnostic value, we further explored the association between ZDHHC12 expression levels and clinical prognosis in pan-cancer patients. Univariate Cox proportional hazards regression analysis indicated that ZDHHC12 expression was associated with prognosis in multiple tumor types. A forest plot clearly illustrated the prognostic landscape of ZDHHC12 as either a risk factor (HR > 1) or a protective factor (HR < 1) across different cancers, with ZDHHC12 demonstrating pronounced prognostic significance in glioma (Fig. 1D), suggesting that the association between ZDHHC12 expression and patient survival may vary across tumor types.

To validate the above transcriptome-based findings, we retrieved immunohistochemical staining data from the HPA database to assess ZDHHC12 expression at the protein level^22^. Stacked bar charts revealed that ZDHHC12 protein displayed varying degrees of high to moderate expression across multiple clinical tumor tissue samples, including glioma, breast cancer, cervical cancer, and colorectal cancer (Fig. 1E). Notably, high ZDHHC12 expression was predominantly enriched in immune subtypes C1, C2, and C3, particularly the C2 subtype (IFN-γ dominant), suggesting a potential association with IFN-γ and CD8^+^ T cells (Fig. 1F).

Given the potential pan-cancer relevance of ZDHHC12 and to preliminarily explore the pathway signatures associated with ZDHHC12 expression, we analyzed the correlation between its expression levels and the combined Z-scores of canonical oncogenic signaling pathways^34^. Correlation scatter plots revealed that ZDHHC12 expression was significantly associated with enrichment scores of key cancer hallmark pathways, including apoptosis, epithelial- mesenchymal transition, and DNA damage repair (Fig. 1G). These findings indicate that ZDHHC12 expression is associated with several cancer-related pathway signatures, including apoptosis, EMT, and DNA damage repair. Further functional studies are required to determine whether ZDHHC12 directly participates in these biological processes.

### 3.2. ZDHHC12 Is Associated with the Pan-Cancer Immune Microenvironment and Antitumor Immunity

**Figure 2:**
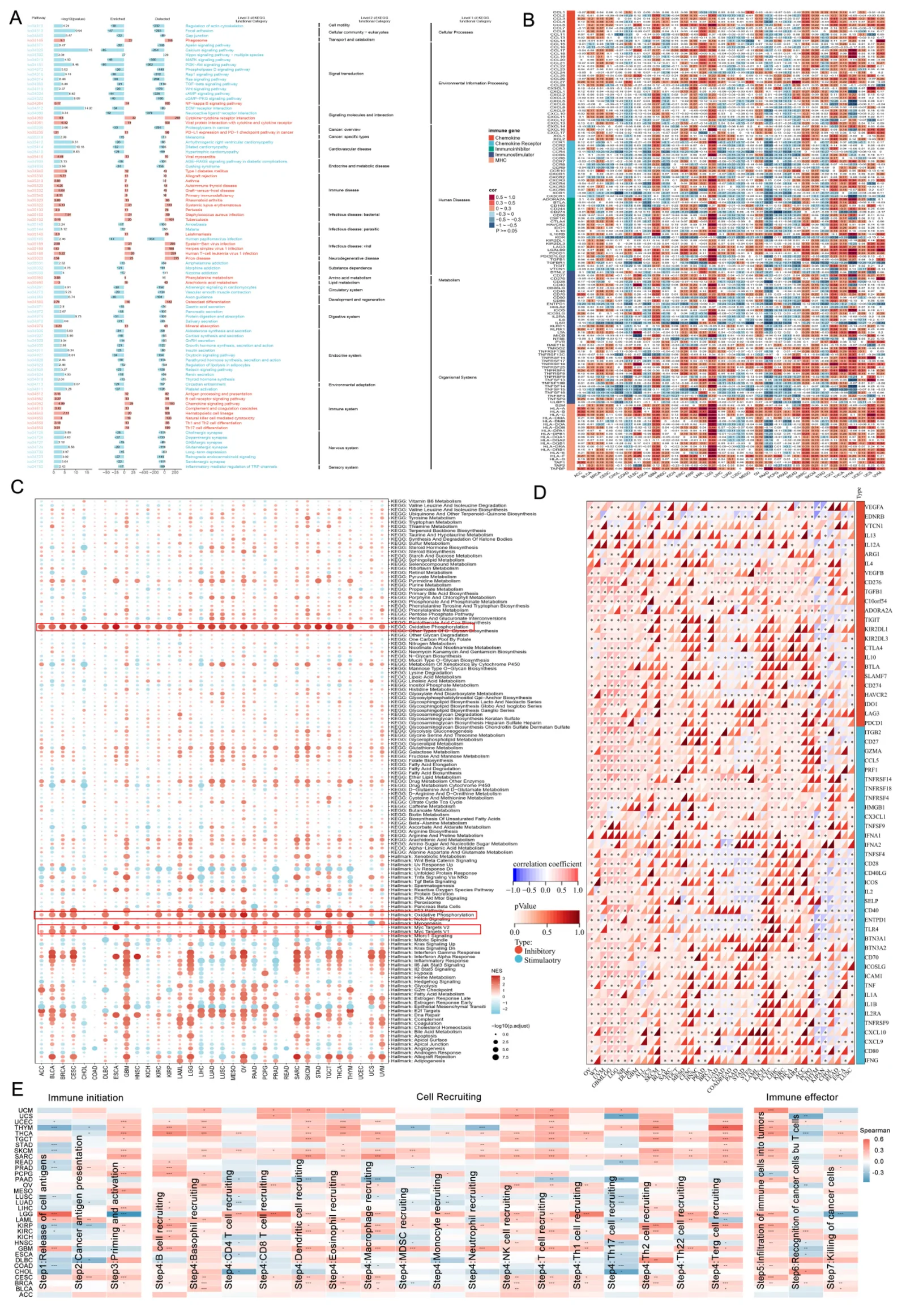
Correlation of ZDHHC12 expression with the pan-cancer immune microenvironment and the antitumor immunity cycle. (A, C) GSEA based on Hallmark and KEGG gene sets. (B) Relationships between ZDHHC12 and various immune-related genes, including chemokines, chemokine receptors, immunosuppressive factors, immunostimulatory factors, and major histocompatibility complex (MHC) molecules. (D) Analysis of the correlation between ZDHHC12 expression levels and immune checkpoints. (E) Feature matrix of a high-resolution tumor immune landscape, illustrating the correlation network of ZDHHC12 within three core modules: immune initiation, cell recruitment, and immune effector functions.

The infiltration characteristics and functional status of immune cells within the TME critically determine tumor immune evasion and patient responses to clinical interventions^35^. To characterize the immune-related features associated with ZDHHC12 expression, we first systematically assessed its biological functions at the pan-cancer level using functional enrichment analysis, which revealed that ZDHHC12 is closely associated with multiple immune pathways (Fig. 2A). Further correlation heatmaps demonstrated significant associations between ZDHHC12 and a wide range of immune-related genes, including chemokines, chemokine receptors, immunosuppressive factors, immunostimulatory factors, and MHC molecules, with particularly strong correlations observed in GBM and LGG (Fig. 2B). These findings indicate that ZDHHC12 is associated with tumor progression, and together with its expression and prognostic features, suggest that ZDHHC12 may be especially important in glioma progression. Subsequent GSEA revealed that ZDHHC12 was significantly enriched in pathways such as oxidative phosphorylation, MYC targets V1, and MYC targets V2 (Fig. 2C), suggesting that ZDHHC12 may be involved in energy metabolism, mitochondrial damage, and cell proliferation. Moreover, ZDHHC12 appeared to act as an immunosuppression/tumor immune evasion-associated gene, as its elevated expression was closely linked to upregulation of immune checkpoint molecules, increased T cell exhaustion markers, and downregulation of T cell activation/killing function genes (Fig. 2D).

To further delineate the role of ZDHHC12 in the dynamic process of antitumor immune responses at high resolution, we introduced the classical cancer immunity cycle framework. This cycle encompasses seven key successive physiological stages, from antigen release to eventual tumor cell killing^36^. By quantitatively calculating the enrichment scores of immune signatures at each stage, we dissected the deeper associations between ZDHHC12 expression and individual immune steps. The results showed that ZDHHC12 expression was associated with signatures representing three major modules of the cancer immunity cycle: immune initiation, immune-cell recruitment, and immune effector activity. During the immune initiation phase, ZDHHC12 expression was correlated with signatures related to tumor antigen release, antigen presentation, and immune-cell priming and activation. During the immune-cell recruitment phase, ZDHHC12 expression was associated with signatures reflecting the recruitment of CD4^+^ T cells, CD8^+^ T cells, natural killer cells, dendritic cells, macrophages, monocytes, neutrophils, and myeloid-derived suppressor cells (Fig. 2E). ZDHHC12 expression was also associated with signatures related to immune-cell infiltration, tumor-cell recognition, and tumor-cell killing. Collectively, these analyses indicate that ZDHHC12 expression is correlated with multiple components of the cancer immunity cycle, although these associations do not establish a direct regulatory role.

### 3.3. Association of ZDHHC12 Expression with Pan-Cancer Microenvironment Components and Antitumor Immunity in Glioma

**Figure 3:**
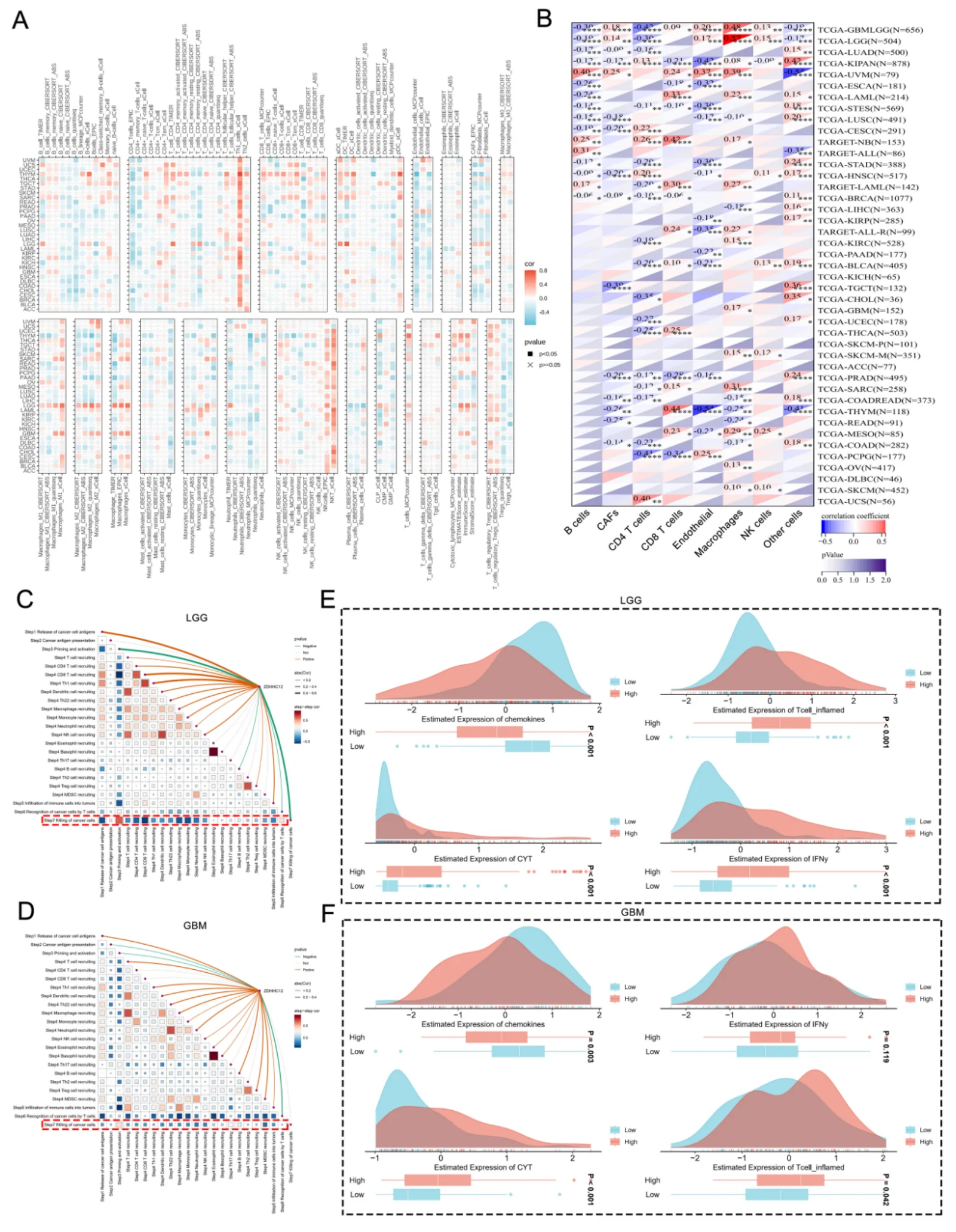
Correlation of ZDHHC12 expression with immune cell infiltration in the TME. (A) Relationship between ZDHHC12 expression levels and immune infiltration in pan-cancer. (B) Correlation analysis between ZDHHC12 expression and immune cells calculated by EPIC. (C, D) Immune cycle analysis of ZDHHC12 in LGG and GBM. (E, F) Comparisons of chemokine, T-cell-inflammation, CYT, and IFN-γ-related scores between the ZDHHC12-high and ZDHHC12-low groups in LGG and GBM.

Tumor tissues represent complex ecosystems composed of malignant cells, stromal cells, and infiltrating immune cells. To quantify the association of ZDHHC12 expression with TME heterogeneity, we first employed the TIMER database to depict the overall landscape of immune cell infiltration. The results showed that in multiple solid tumors, including lower-grade glioma (LGG) and GBM, ZDHHC12 expression levels were associated with lower CD8^+^ T cell infiltration and higher Th1 and Th2 cell infiltration, indicating an association with altered immune-infiltration patterns (Fig. 3A). Immune cell analysis further revealed that ZDHHC12 was closely correlated with immune infiltration levels across various cancers. Notably, among all immune subsets analyzed, macrophage infiltration abundance exhibited the most consistent and strong positive correlation with ZDHHC12 expression, particularly pronounced in glioma (Fig. 3B).

Given that TAMs play a central driving role in the formation of the immunosuppressive microenvironment, tumor invasion, and therapeutic resistance in glioma, this strong positive correlation provided a critical clue to the potential biological relevance of ZDHHC12. Cancer immunity cycle analysis indicated that in LGG and GBM, ZDHHC12 was positively correlated with antigen release; however, high ZDHHC12 expression was associated with lower tumor-cell-killing scores and altered immune-infiltration-related signatures (Fig. 3C, 3D). To further investigate the association between ZDHHC12 expression and local immune features across glioma subtypes, we quantitatively assessed the association between ZDHHC12 expression and four core immune microenvironment features, including chemokines, T cell inflammation, cytolytic activity (CYT), and IFN-γ in glioma cohorts. In the LGG cohort, CYT, IFN-γ, and T-cell-inflammation scores were all significantly higher in the ZDHHC12-high group than in the ZDHHC12-low group (all P < 0.001). However, in stark contrast to the active inflammatory signals, chemokine scores were markedly downregulated (P < 0.001) (Fig. 3E). In GBM, chemokine, CYT, and T-cell-inflammation scores also differed between the ZDHHC12-high and -low groups, whereas the difference in the IFN-γ-related score did not reach statistical significance (Fig. 3F). Collectively, these results indicate that high ZDHHC12 expression is associated with distinct immune microenvironmental features in glioma; however, the underlying cellular mechanisms cannot be determined from these correlation analyses.

### 3.4. Single-Cell and Spatial Transcriptomics Reveal Cellular Heterogeneity of ZDHHC12 and Its Co-localization with Macrophages in Glioma

**Figure 4:**
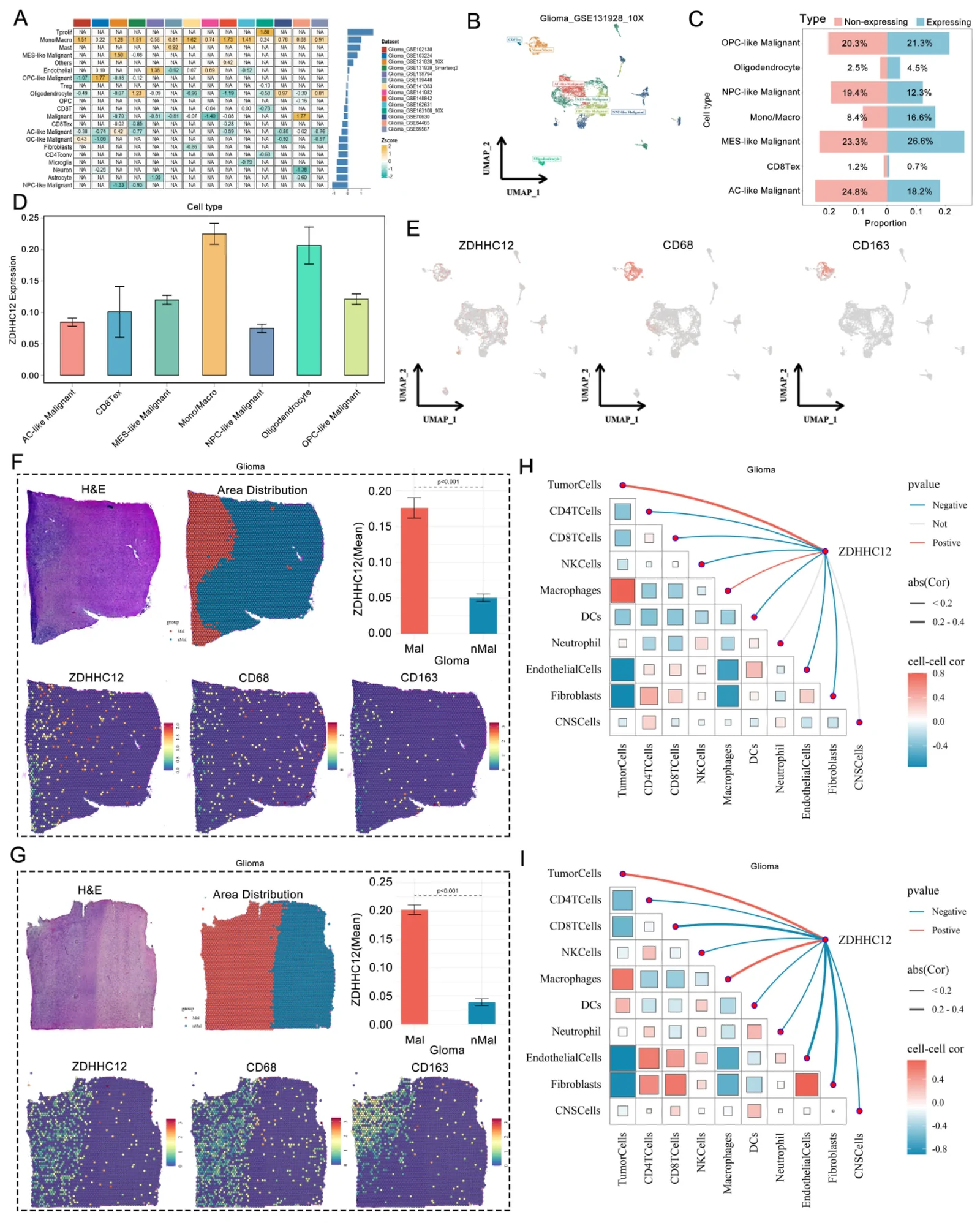
Single-cell and spatial transcriptomic analysis reveals cellular heterogeneity of ZDHHC12 and its spatial co-localization with macrophages in glioma. (A) Expression atlas of ZDHHC12 in the glioma single-cell transcriptome cohort. (B) Expression distribution characteristics of cell subpopulations and malignant tumor cell subtypes in the single-cell dataset. (C) Differential expression of ZDHHC12 across various cell subpopulations in the single-cell atlas. (D) Single-cell feature plots showing the expression of ZDHHC12. (E) UMAP plots showing the expression of ZDHHC12 and the general macrophage marker CD68 and the immunoregulatory macrophage-associated marker CD163. (F, G) Spatial transcriptomic analysis of glioma. Includes H&E-stained images, spatial annotations of malignant (Mal) and non-malignant (nMal) regions, and spatial feature plots demonstrating the co-localization of ZDHHC12 with CD68 and CD163. Bar plots show quantitative expression differences of ZDHHC12 between Mal and nMal regions. (H, I) Assessment of correlations between gene expression levels and cell type proportions.

Given the strong positive correlation between ZDHHC12 expression and macrophage infiltration observed in pan-cancer microenvironment analyses, we further focused on glioma-a tumor characterized by pronounced heterogeneity and an immunosuppressive microenvironment-to characterize its cellular distribution and spatial associations using high resolution single-cell transcriptomic data. First, multiple glioma single-cell datasets revealed that ZDHHC12 was significantly enriched in monocyte/macrophage populations (Fig. 4A). Through unsupervised clustering and nonlinear dimensionality reduction (UMAP) of glioma single-cell sequencing data, we successfully partitioned the complex TME into distinct cell subsets and characterized the expression distribution of ZDHHC12 across these subsets. Within malignant cell populations, glioma cells were further classified into neural progenitor cell-like (NPC-like), oligodendrocyte progenitor cell-like (OPC-like), astrocyte-like (AC-like), and mesenchymal-like (MES-like) states based on established classifiers^2^. Statistical analysis demonstrated that ZDHHC12 was most significantly enriched in monocyte/macrophage clusters (Fig. 4B, 4D). Moreover, within the monocyte/macrophage subsets, ZDHHC12 exhibited the greatest variation in expression proportion (Fig. 4C). Single-cell feature plots revealed substantial overlap between ZDHHC12 expression and clusters expressing the general macrophage marker CD68 and the immunoregulatory macrophage-associated marker CD163 (Fig. 4E). These findings suggest that ZDHHC12 may serve as a potential biomarker within glioma macrophage subsets and may be associated with macrophage-related immune features in the glioma microenvironment.

Although single-cell transcriptomics provides unprecedented resolution, it loses critical spatial positional information during tissue dissociation. To validate the above findings within an intact tissue architecture, we incorporated glioma spatial transcriptomic data. By high-precision alignment of H&E-stained pathological sections with spatial gene expression matrices, tissue sections were rigorously divided into malignant and non-malignant regions (Fig. 4F, 4G). Spatial transcriptomic data revealed that the mean expression level of ZDHHC12 was significantly higher in malignant regions than in non-malignant regions (P < 0.001). Furthermore, spatial feature distribution maps visually demonstrated a high degree of topological concordance between ZDHHC12 and CD68, as well as the immunoregulatory macrophage-associated marker CD163 (Fig. 4F, 4G). To further characterize the relationships between ZDHHC12 expression and cellular composition in the TME, we constructed correlation networks between gene expression levels and inferred cell-type proportions (Fig. 4H, 4I). The network analyses showed that ZDHHC12 expression was correlated with malignant-cell and macrophage-related features, supporting its potential value as a marker associated with TAM abundance and macrophage-related transcriptional states.

### 3.5. Associations among ZDHHC12, BAK1, and IFNB1 in the Glioma Immune Microenvironment

**Figure 5:**
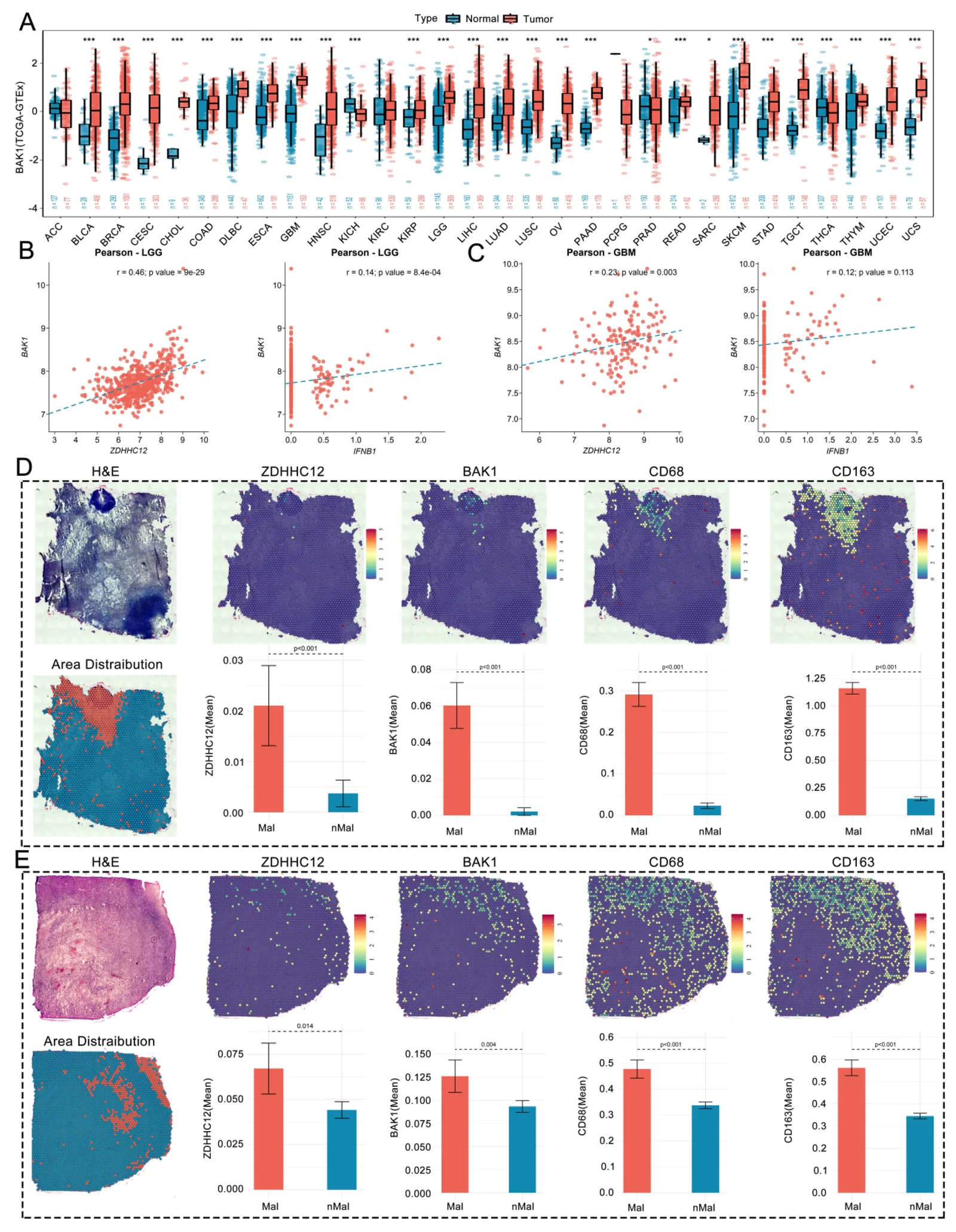
Expression correlation of ZDHHC12 and BAK1 in glioma and their spatial co-localization with macrophages. (A) Pan-cancer mRNA differential expression analysis of the pro-apoptotic gene BAK1 based on the TCGA and GTEx databases. (B, C) Correlation analyses showing the relationships between ZDHHC12 and BAK1 expression and between BAK1 and IFNB1 expression in LGG and GBM. (D, E) Spatial transcriptomic analysis revealing spatial co-localization of ZDHHC12, BAK1, and macrophage markers (CD68, CD163). Subpanels include H&E-stained images, histological annotations of malignant (Mal) and non-malignant (nMal) regions, spatial feature plots, and bar plots quantitatively comparing the expression differences of each target gene between Mal and nMal regions.

To identify molecules potentially associated with ZDHHC12 and macrophage-related features in the glioma microenvironment, we screened genes whose expression was strongly correlated with ZDHHC12 in TCGA glioma cohorts. BAK1 was subsequently selected as a candidate ZDHHC12-associated molecule for further correlation and spatial analyses. Among these, BCL2 antagonist/killer 1 (BAK1) is a classical key pro-apoptotic and metabolic regulatory protein. Pan-cancer transcriptomic profiling revealed that BAK1 also exhibited significant differential expression across multiple malignancies, including glioma, closely mirroring the pan-cancer expression landscape of ZDHHC12 (Fig. 5A).

We subsequently performed co-expression analyses of ZDHHC12 with BAK1 and of BAK1 with IFNB1. The results showed that BAK1 was significantly positively correlated with ZDHHC12 in both LGG and GBM (Fig. 5B, 5C). Additionally, BAK1 expression was positively correlated with IFNB1 expression in LGG, whereas this association did not reach statistical significance in GBM (Fig. 5B, 5C).

Although bulk RNA-seq analysis revealed significant correlations, we further examined spatial transcriptomic data to determine whether ZDHHC12 and BAK1 exhibited spatial associations with macrophage markers within intact glioma tissue. The results demonstrated that the average expression levels of ZDHHC12, BAK1, CD68 and CD163 all showed significantly higher expression in malignant regions (Fig. 5D, 5E). These findings demonstrate spatial associations among ZDHHC12, BAK1, CD68, and CD163 in malignant glioma regions but do not establish direct molecular interactions or functional causality.

### 3.6. Pathway Enrichment and Immune Microenvironmental Features Associated with ZDHHC12 Expression in Glioma

**Figure 6:**
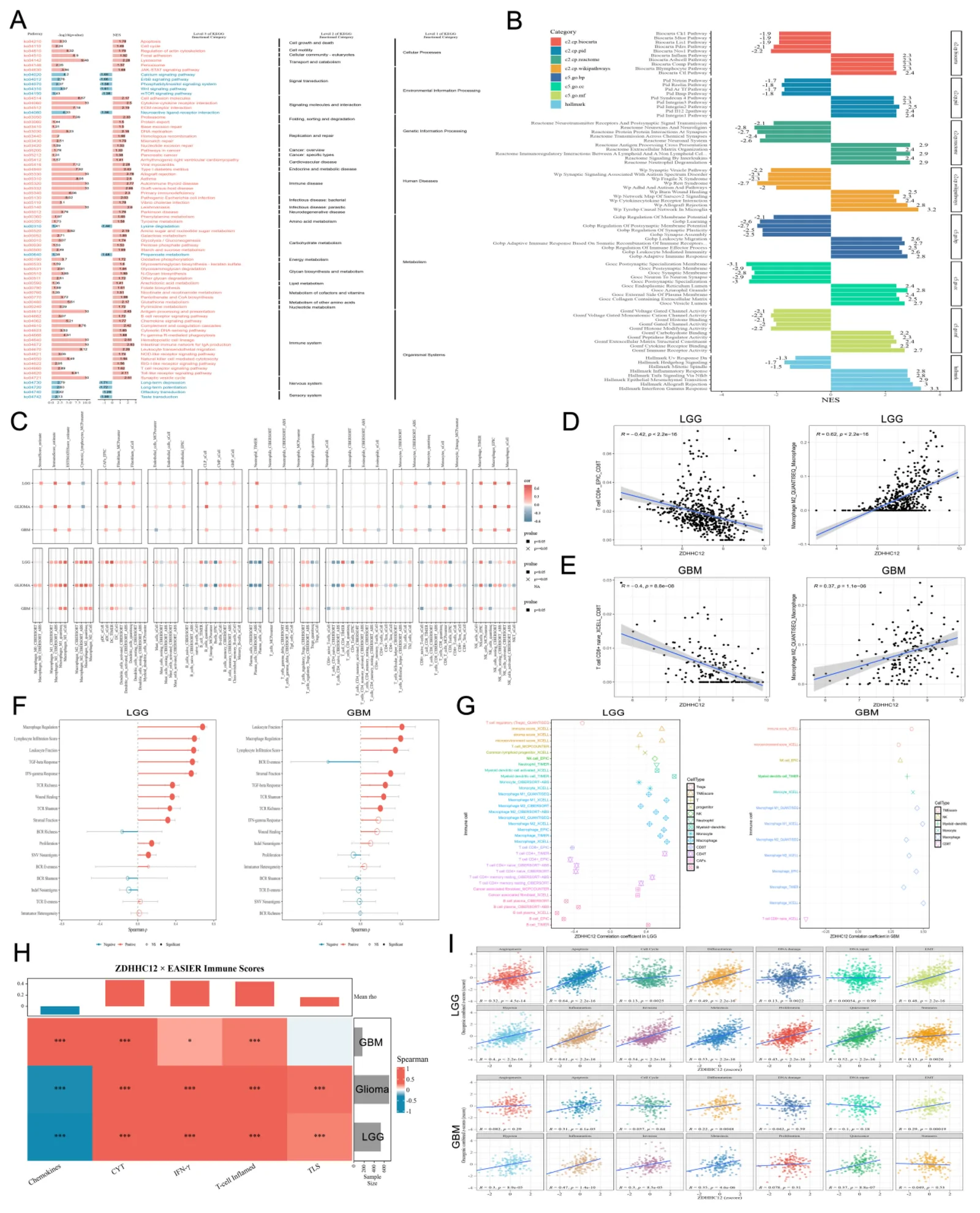
Pathway enrichment and immune microenvironmental features associated with ZDHHC12 expression in glioma. (A) KEGG functional enrichment analysis associated with ZDHHC12 in the glioma cohort. (B) Enrichment analysis associated with ZDHHC12 in the glioma cohort. (C) Immune cell infiltration analysis based on ZDHHC12 expression. (D) Correlation analysis of ZDHHC12 expression with T cell and macrophage infiltration in LGG. (E) Correlation analysis of ZDHHC12 expression with T cell and macrophage infiltration in GBM. (F) Spearman correlation analysis between ZDHHC12 expression and immune-related functional signatures in LGG and GBM. (G) Correlations between ZDHHC12 expression and microenvironment components. (H) EaSIeR-based correlation analyses showing the associations of ZDHHC12 expression with IFNγ-related, T-cell-inflammation, chemokine, and tertiary lymphoid structure signatures in LGG and GBM. (I) Correlation between ZDHHC12 expression and malignant hallmarks of tumors.

To characterize the biological pathways and immune microenvironmental features associated with ZDHHC12 expression at the transcriptome-wide level, we performed GO and KEGG enrichment analyses using the TCGA glioma cohort. KEGG pathway analysis revealed that differentially expressed genes were highly enriched in "cytokine cytokine receptor interaction," "phagosome," and multiple immune-related pathways (Fig. 6A). Enrichment analysis further demonstrated that aberrant ZDHHC12 expression was similarly enriched in immune-related biological processes (Fig. 6B). These enrichment results were consistent with the single-cell and spatial transcriptomic findings showing associations of ZDHHC12 with macrophage-related features and BAK1 expression.

Although conventional enrichment analysis revealed significantly associated biological modules; to further evaluate the potential value of ZDHHC12 in immune cell infiltration in glioma, we performed immune infiltration analysis. The results showed that ZDHHC12 was significantly correlated with the infiltration levels of multiple immune cell types in glioma, with multiple algorithms consistently indicating a particularly strong association between ZDHHC12 and macrophage infiltration (Fig. 6C). Subsequent correlation scatter plots demonstrated that in LGG, ZDHHC12 was significantly positively correlated with macrophage infiltration but significantly negatively correlated with CD8^+^ T cell infiltration (Fig. 6D). Similar results were validated in GBM (Fig. 6E). Furthermore, Spearman correlation analysis revealed significant associations between ZDHHC12 expression and multiple macrophage- and immune-related functional signatures in LGG and GBM (Fig. 6F). Spearman correlation analysis further revealed that high ZDHHC12 was positively correlated with macrophage infiltration and negatively correlated with CD8^+^ T-cell infiltration (Fig. 6G).

Multi-cohort correlation analyses based on the EaSIeR framework showed that ZDHHC12 expression was associated with IFN-γ-related, T-cell-inflammation, chemokine, and tertiary lymphoid structure signatures in glioma. The direction and magnitude of these associations differed between LGG and GBM. In LGG, ZDHHC12 expression was negatively correlated with chemokine-related scores, whereas in GBM it showed a positive correlation with chemokine-related features and a weaker association with tertiary lymphoid structure signatures. These subtype-specific correlations suggest that the immune context associated with ZDHHC12 expression may differ between LGG and GBM; however, mechanistic interpretation requires further experimental validation (Fig. 6H).

Collectively, these analyses showed that ZDHHC12 expression was associated with multiple malignant phenotypes and immune microenvironmental features in glioma (Fig. 6I). In particular, ZDHHC12 expression was positively correlated with macrophage infiltration and negatively correlated with CD8^+^ T-cell infiltration. These findings support further investigation of ZDHHC12 as a candidate biomarker of macrophage-enriched and immunosuppressive features in glioma.

### 3.7. Drug Sensitivity Associations and Candidate Compound Screening Based on ZDHHC12 Expression

Previous multi-omics analyses have established ZDHHC12 as a molecule associated with macrophage-related and immunosuppressive features. Despite the increasing diversification of therapeutic modalities, resistance to chemotherapy and radiotherapy remains a major challenge in conventional treatment^37^. Emerging evidence indicates that the development of drug resistance in tumors is associated with aberrant gene regulation^38^-^40^. To determine whether ZDHHC12 expression is associated with drug-response metrics, we analyzed its association with drug sensitivity.

**Figure 7:**
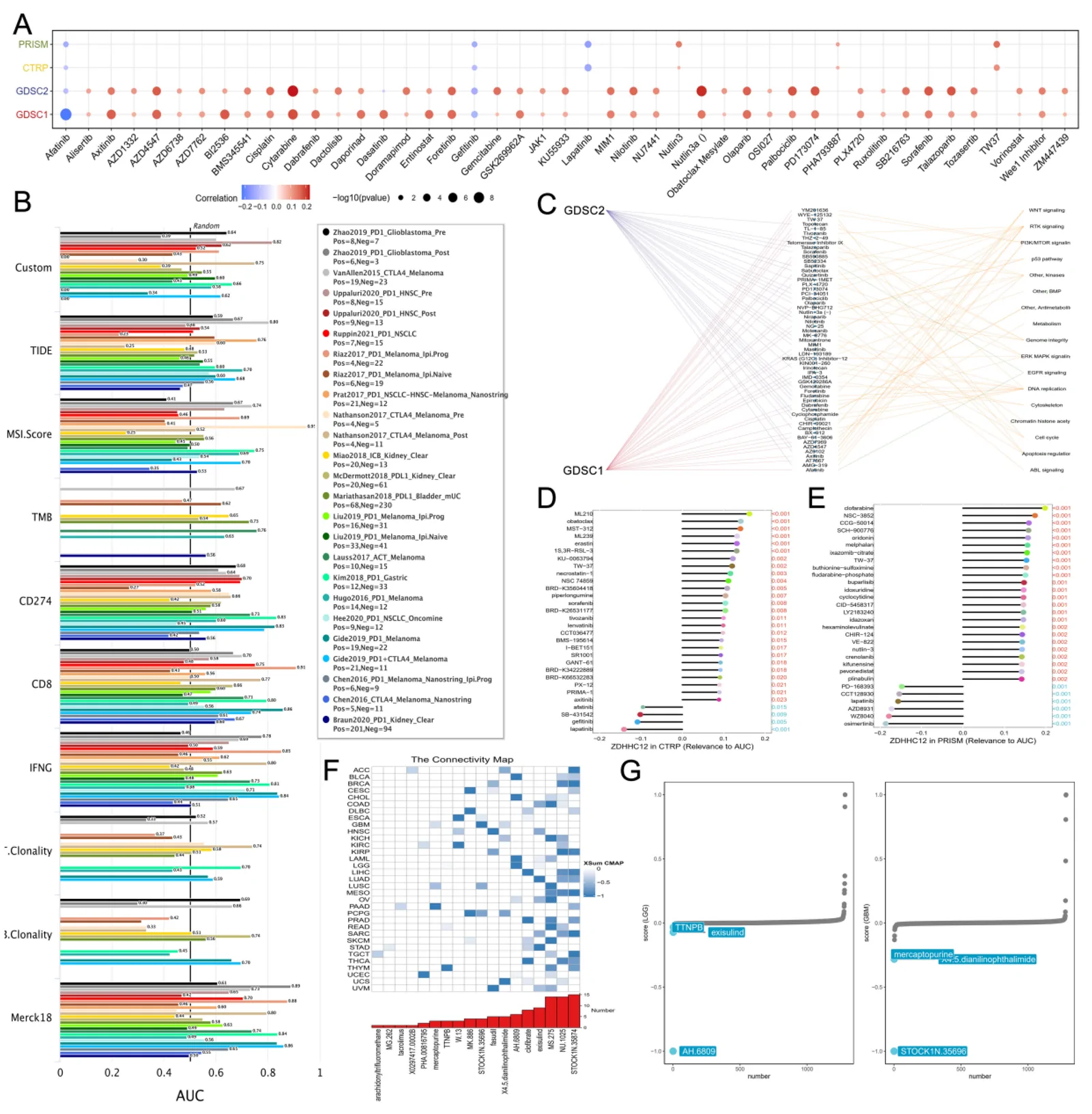
Drug sensitivity associations and computational screening of candidate compounds related to ZDHHC12 expression. (A) Correlations between ZDHHC12 expression and drug response metrics based on data from the PRISM, GDSC1, GDSC2, and CTRP databases. (B) Associations between ZDHHC12 expression and immunotherapy-related response metrics across immune checkpoint blockade cohorts based on the TIDE database. (C) The top 30 drugs and their mechanism pathways in two databases. (D) Spearman correlation analysis between ZDHHC12 expression and drug response AUC values in the CTRP database. (E) Spearman correlation analysis between ZDHHC12 expression and drug response AUC values in the PRISM database. (F) Associations between various compounds and pan-cancer cells. (G) Candidate compounds computationally predicted by the XSum algorithm to reverse ZDHHC12-associated transcriptional signatures.

First, to systematically evaluate the relationship between ZDHHC12 expression and the efficacy of conventional chemotherapeutic and targeted agents, we performed comprehensive analyses using four databases. Results from the GDSC1 and GDSC2 databases revealed that ZDHHC12 exhibited strong positive correlations with the IC50 values of multiple drugs (Fig. 7A), indicating an association between ZDHHC12 expression and predicted drug-response metrics. To characterize the pathways targeted by the identified compounds, we mapped ZDHHC12 associated drugs to their corresponding signaling pathways. These drugs were predominantly enriched in receptor tyrosine kinase (RTK), phosphoinositide 3-kinase/mechanistic target of rapamycin (PI3K/mTOR), and mitogen-activated protein kinase (MAPK) signaling pathways, as well as processes related to DNA replication, cell cycle, and apoptosis (Fig. 7C).

Using the TIDE database, we evaluated ZDHHC12 associated antitumor immune responses by inputting 25 immune checkpoint blockade treatment sub-cohorts and genes previously reported to be involved in tumor immune evasion for comparative analysis with ZDHHC12. The results demonstrated that ZDHHC12 showed variable associations with immunotherapy-related response metrics across different cohorts (Fig. 7B). The CTRP and PRISM databases further revealed associations between ZDHHC12 overexpression and drug-response measures for several therapeutic compounds (Fig. 7D, E). Additionally, the Extreme Sum (XSum) algorithm combined with the Connectivity Map (CMap) database was employed to screen for candidate compounds for future preclinical screening. The results showed that STOCKN.35874, NU.1025, and MS.275 were the top three candidate drugs based on differential ZDHHC12 expression levels (Fig. 7F). Furthermore, AH.6809 and STOCK1N.35696 were computationally identified as candidate compounds potentially associated with reversal of ZDHHC12-related transcriptional signatures in LGG and GBM, respectively (Fig. 7G).

Collectively, these analyses identify associations between ZDHHC12 expression and predicted responses to several therapeutic agents. The candidate compounds identified through pharmacogenomic databases and computational algorithms should be regarded as a hypothesis-generating resource for future preclinical screening rather than as evidence supporting immediate clinical use. Further validation in glioma cell models, patient-derived organoids, in vivo models, and independent treatment cohorts is required to determine their biological and therapeutic relevance.

## 4. Discussion

With the continuous advancement of precision oncology, dissecting the heterogeneity of the TME and identifying novel biomarkers has become critical for overcoming cancer recurrence and therapeutic resistance. Protein S-palmitoylation mediated by the ZDHHC family exerts profound effects on oncogenic signaling and microenvironmental homeostasis^19^. However, the pan-cancer expression landscape and immune microenvironmental associations of ZDHHC12, particularly in glioma, remain incompletely characterized. In this study, we integrated bulk RNA-seq, single-cell transcriptomic, spatial transcriptomic, immune infiltration, and pharmacogenomic analyses to characterize ZDHHC12 across cancer types. Our findings consistently showed associations between ZDHHC12 expression and macrophage-enriched, immunosuppressive, and treatment-response-related features in glioma. These observations are hypothesis-generating and do not establish direct molecular or cellular causality.

First, our pan-cancer landscape analysis confirmed that ZDHHC12 is significantly overexpressed in multiple malignancies, including glioma, and exhibits excellent diagnostic performance in distinguishing tumor from normal tissues. ZDHHC12 expression was also associated with prognosis in several cancer types, although multivariable analyses and independent clinical validation are required to determine whether it represents an independent prognostic factor. To further characterize the biological and immune microenvironmental features associated with ZDHHC12 expression, we focused on the tumor immune microenvironment (TME). Analysis of the cancer immunity cycle showed that ZDHHC12 expression was associated with signatures representing antigen presentation, immune-cell recruitment, and tumor-cell-killing activity^36^. Transcriptomic analysis of multiple ovarian cancer datasets, combined with data from the SNU119 high-grade serous ovarian cancer model, indicated that elevated ZDHHC12 expression is associated with enhanced activity of DNA damage response pathways, further supporting its potential biological relevance. Consistent with these findings, previous studies have shown that inhibition of ZDHHC12 increases sensitivity to cisplatin^41^, suggesting that excessively high ZDHHC12 activity may broadly attenuate tumor responses to DNA damage-based therapies. Furthermore, macro scale microenvironment deconvolution analysis revealed that ZDHHC12 expression is strongly positively correlated with the infiltration levels of stromal and innate immune components, including macrophages and cancer associated fibroblasts. Collectively, these findings support further investigation of ZDHHC12 as a candidate biomarker associated with TME.

Currently, the role of ZDHHC12 in cancer remains incompletely understood. Previous studies have linked ZDHHC12 to cisplatin resistance in high-grade serous ovarian cancer^41^, ovarian cancer progression through claudin-3 (CLDN3) S-palmitoylation, and glioma progression^42^,^43^. On the other hand, ZDHHC12 mediated palmitoylation of NLRP3 suppresses inflammatory responses, suggesting a potential tumor suppressive role under certain contexts, although direct evidence in cancer is lacking^19^. Nevertheless, the specific involvement of ZDHHC12 in pan cancer and glioma immune microenvironment remodeling remains underexplored. Given that gliomas (LGG/GBM) are characterized by extreme cellular heterogeneity and an immunosuppressive microenvironment rich in TAMs^44^, elucidating the underlying regulatory mechanisms is of great scientific significance. A recent study demonstrated that ZDHHC12 deficiency in macrophages significantly reduces the palmitoylation of mitochondrial antiviral-signaling protein (MAVS) and RNA virus-triggered type I interferon signaling in THP-1-derived macrophages and bone marrow-derived macrophages^45^.

In this study, we leveraged high resolution multi-omics approaches to overcome the limitations of conventional bulk analyses, which are confounded by cellular heterogeneity in tissue samples. Single-cell transcriptomic analysis characterized the distribution of ZDHHC12 across malignant and immune cell populations and showed comparatively high expression in monocyte/macrophage subsets. More importantly, in situ validation using spatial transcriptomics filled the gap left by single-cell technology regarding the loss of spatial topological information. Spatial feature maps showed that hotspots of ZDHHC12 high expression in malignant regions exhibited substantial spatial overlap with CD68/CD163 expressing patches. This high degree of spatial concordance strongly suggests that ZDHHC12 may be associated with local monocyte/macrophage accumulation and CD163-positive macrophage-related states.

Differential expression of palmitoylation related genes, particularly ZDHHC5, ZDHHC12, LYPLA1, and PPT2, has suggested their potential roles in the pathogenesis and prognosis of lung adenocarcinoma^46^. ZDHHC12 has been demonstrated to be closely associated with tumor progression. Studies have shown that ZDHHC12 mediated palmitoylation of Claudin-3 plays a decisive role in ovarian cancer progression^42^. Furthermore, ZDHHC12 mediated palmitoylation of specific proteins affects the response of high-grade serous ovarian cancer to cisplatin through reactive oxygen species-related mechanisms^41^. To explore molecules associated with ZDHHC12 expression, we identified a significant transcriptional and spatial correlation between ZDHHC12 and the pro-apoptotic factor BAK1. BAK1, a key member of the Bcl2 family, is traditionally recognized as a core mediator of the mitochondrial apoptosis pathway. Interestingly, we observed a strong positive correlation between ZDHHC12 and BAK1 at the transcriptional level in glioma, and spatial transcriptomic data showed substantial spatial overlap in malignant regions. GSEA results showed that high ZDHHC12 expression was associated with enrichment of apoptosis-, epithelial-mesenchymal transition-, leukocyte migration-, macrophage activation-, and IL6-JAK-STAT3-related signatures. We hypothesize that ZDHHC12 may be functionally linked to BAK1-related apoptotic processes in glioma. However, whether ZDHHC12 directly palmitoylates BAK1 or alters its localization, stability, or activity remains unknown and requires experimental validation.

Tumor-infiltrating immune cells profoundly influence cancer treatment outcomes and patient prognosis^47^. To date, studies on the relationship between ZDHHC12 and tumor immune infiltration remain limited. Because ZDHHC12 expression was correlated with macrophage infiltration, CD8^+^ T-cell infiltration, immune checkpoint expression, and pharmacogenomic response metrics, it may reflect variation in the immune and therapeutic response landscape of glioma. However, these findings were derived from computational analyses and should not be interpreted as evidence that ZDHHC12 directly regulates cytokine production, immune-cell recruitment, drug resistance, or immunotherapy response. In particular, CD163 expression alone is insufficient to define a definitive M2-like macrophage phenotype, and CD68 is a general macrophage marker rather than an M2-specific marker. Therefore, the observed co-expression and spatial co-localization of ZDHHC12 with CD68 and CD163 should be interpreted as an association with macrophage-rich and potentially immunoregulatory regions. The associations between ZDHHC12 expression and drug response metrics in GDSC, CTRP, PRISM, CMap, and TIDE provide a hypothesis-generating resource for future studies. The identified compounds should be regarded as candidates for preclinical screening rather than clinically actionable treatment recommendations. Validation in glioma cell lines, patient-derived cells or organoids, in vivo models, and independent clinical treatment cohorts is required before the prognostic or therapeutic utility of ZDHHC12 can be established.

Despite the comprehensive integration of multiple public datasets, several limitations should be acknowledged. First, the study was based primarily on retrospective data from public databases, and independent prospective clinical cohorts are required to validate the prognostic associations of ZDHHC12. Second, the single-cell, spatial transcriptomic, and immune infiltration analyses were observational and correlational. They do not establish that ZDHHC12 directly regulates macrophage recruitment, polarization, or immune suppression. Third, CD163 expression alone is insufficient to define a definitive M2-like macrophage phenotype, and CD68 is not an M2-specific marker. Comprehensive macrophage signature scoring, protein-level validation, and functional phenotyping are therefore required. Finally, the drug sensitivity and immunotherapy-related results were derived from computational prediction models and have not been validated in independent glioma treatment cohorts or experimental models. Future studies involving ZDHHC12 knockdown or overexpression, glioma-macrophage co-culture and chemotaxis assays, multidimensional macrophage phenotyping, patient-derived models, and in vivo glioma models are required to establish causality and therapeutic relevance.

## 5. Conclusion

In summary, integrated pan-cancer, single-cell, and spatial transcriptomic analyses identified ZDHHC12 as a candidate biomarker associated with prognosis across multiple cancers. In glioma, ZDHHC12 expression was associated with macrophage infiltration, CD68/CD163-positive spatial regions, BAK1 expression, and immunosuppressive microenvironmental features. These findings provide a basis for further investigation, although functional and experimental studies are required to establish causality and determine the clinical relevance of ZDHHC12.

## Data Availability

Publicly available datasets were analyzed in this study. TCGA data were obtained from the TCGA database, GTEx data from the GTEx Portal, protein expression data from the Human Protein Atlas, and single-cell and spatial transcriptomic data from the Gene Expression Omnibus. Drug response and immunotherapy-related data were obtained from GDSC, CTRP, PRISM, CMap, and TIDE.

## Abbreviations

TME: Tumor microenvironment
TAMs: Tumor-associated macrophages
TCGA: The Cancer Genome Atlas
GTEx: Genotype-Tissue Expression
HPA: Human Protein Atlas
GSVA: Gene set variation analysis
ROC: Receiver operating characteristic
AUC: Area under the curve
OS: Overall survival
GSEA: Gene Set Enrichment Analysis
GO: Gene Ontology
KEGG: Kyoto Encyclopedia of Genes and Genomes
CESC: Cervical squamous cell carcinoma and endocervical adenocarcinoma
UCEC: Uterine corpus endometrial carcinoma
GBM: Glioblastoma
LGG: lower-grade glioma
EMT: Epithelial-mesenchymal transition
ssGSEA: single-sample gene set enrichment analysis
XSum: Extreme Sum

## References

[1] Yang N, Yuan H, Wang W (2026). Pan-cancer macrophage atlas uncovers that MHC-II(high) TAMs induce Treg activation through physical interactions. Cell Rep.

[2] Zhang C, Hu B, Wang C, Hou S, Lin N (2025). Unveiling the novel value of TREM1 in the proneural-mesenchymal transition of glioma via tumor-associated macrophages. Front Immunol.

[3] Zhang C, Hou S, Hu B (2025). Self-Assembling Nanoparticles Orchestrate Cuproptosis-Immunotherapy Synergy to Suppress Postoperative Glioma Recurrence. ACS Appl Mater Interfaces.

[4] Wang Y, Wang S, Wang Y (2024). Glucose regulates the HMGB1 signaling pathway through SIRT1 in glioma. Cell Signal.

[5] Li D, Cui G, Yang K (2026). Inhibiting macrophage-derived lactate transport restores cGAS-STING signalling and enhances antitumour immunity in glioblastoma. Nat Cell Biol.

[6] Zhou F, Tao J, Gou H (2024). FSTL1 sustains glioma stem cell stemness and promotes immunosuppressive macrophage polarization in glioblastoma. Cancer Lett.

[7] Marx V (2021). Method of the Year: spatially resolved transcriptomics. Nat Methods.

[8] Chakrabandhu K, Herincs Z, Huault S (2007). Palmitoylation is required for efficient Fas cell death signaling. EMBO J.

[9] Won SJ, Cheung See Kit M, Martin BR (2018). Protein depalmitoylases. Crit Rev Biochem Mol Biol.

[10] Jin X, Hong Y, Zhao Y (2025). ZDHHC12 Palmitoylates HDAC8 to Promote the Progression of Hepatocellular Carcinoma Associated with a Diet High in Saturated Fatty Acids. Adv Sci (Weinh).

[11] Smotrys JE, Linder ME (2004). Palmitoylation of intracellular signaling proteins: regulation and function. Annu Rev Biochem.

[12] Main A, Fuller W (2022). Protein S-Palmitoylation: advances and challenges in studying a therapeutically important lipid modification. FEBS J.

[13] Runkle KB, Kharbanda A, Stypulkowski E (2016). Inhibition of DHHC20-Mediated EGFR Palmitoylation Creates a Dependence on EGFR Signaling. Mol Cell.

[14] Chen X, Ma H, Wang Z (2017). EZH2 Palmitoylation Mediated by ZDHHC5 in p53-Mutant Glioma Drives Malignant Development and Progression. Cancer Res.

[15] Zhang Z, Li X, Yang F (2021). DHHC9-mediated GLUT1 S-palmitoylation promotes glioblastoma glycolysis and tumorigenesis. Nat Commun.

[16] Moon GH, Lee MY, Koh J (2026). Adipocyte-Derived Palmitic Acid Promotes Breast Cancer Malignancy via ZDHHC15-Mediated S-Palmitoylation of PPARgamma. Cancer Sci.

[17] Wang Y, Chen Z, Li R (2025). S-palmitoylation of c-MET by CK2alpha-mediated zDHHC15 phosphorylation drives glioblastoma stem cell tumorigenicity. Neuro Oncol.

[18] Zhang X, Liu Y, Liao X (2026). Targeting ZDHHC12-mediated PARP1 palmitoylation potentiates PARP inhibitor cytotoxicity. Cell Rep.

[19] Wang L, Cai J, Zhao X (2023). Palmitoylation prevents sustained inflammation by limiting NLRP3 inflammasome activation through chaperone-mediated autophagy. Mol Cell.

[20] Cancer Genome Atlas Research N, Weinstein JN, Collisson EA (2013). The Cancer Genome Atlas Pan-Cancer analysis project. Nat Genet.

[21] Consortium GT (2013). The Genotype-Tissue Expression (GTEx) project. Nat Genet.

[22] Uhlen M, Fagerberg L, Hallstrom BM (2015). Proteomics. Tissue-based map of the human proteome. Science.

[23] Yoshihara K, Shahmoradgoli M, Martinez E (2013). Inferring tumour purity and stromal and immune cell admixture from expression data. Nat Commun.

[24] Newman AM, Liu CL, Green MR (2015). Robust enumeration of cell subsets from tissue expression profiles. Nat Methods.

[25] Stuart T, Butler A, Hoffman P (2019). Comprehensive Integration of Single-Cell Data. Cell.

[26] Korsunsky I, Millard N, Fan J (2019). Fast, sensitive and accurate integration of single-cell data with Harmony. Nat Methods.

[27] Yu G, Wang LG, Han Y, He QY (2012). clusterProfiler: an R package for comparing biological themes among gene clusters. OMICS.

[28] Yuan H, Yan M, Zhang G (2019). CancerSEA: a cancer single-cell state atlas. Nucleic Acids Res.

[29] Hanzelmann S, Castelo R, Guinney J (2013). GSVA: gene set variation analysis for microarray and RNA-seq data. BMC Bioinformatics.

[30] Li T, Fu J, Zeng Z (2020). TIMER2.0 for analysis of tumor-infiltrating immune cells. Nucleic Acids Res.

[31] Thorsson V, Gibbs DL, Brown SD (2018). The Immune Landscape of Cancer. Immunity.

[32] Geeleher P, Cox N, Huang RS (2014). pRRophetic: an R package for prediction of clinical chemotherapeutic response from tumor gene expression levels. PLoS One.

[33] Maeser D, Gruener RF, Huang RS (2021). oncoPredict: an R package for predicting in vivo or cancer patient drug response and biomarkers from cell line screening data. Brief Bioinform.

[34] Sanchez-Vega F, Mina M, Armenia J (2018). Oncogenic Signaling Pathways in The Cancer Genome Atlas. Cell.

[35] Binnewies M, Roberts EW, Kersten K (2018). Understanding the tumor immune microenvironment (TIME) for effective therapy. Nat Med.

[36] Chen DS, Mellman I (2013). Oncology meets immunology: the cancer-immunity cycle. Immunity.

[37] Sun Y (2015). Translational horizons in the tumor microenvironment: harnessing breakthroughs and targeting cures. Med Res Rev.

[38] Aissa AF, Islam A, Ariss MM (2021). Single-cell transcriptional changes associated with drug tolerance and response to combination therapies in cancer. Nat Commun.

[39] Han K, Chen S, Chen WH (2013). Synergistic gene and drug tumor therapy using a chimeric peptide. Biomaterials.

[40] Yang W, Soares J, Greninger P (2013). Genomics of Drug Sensitivity in Cancer (GDSC): a resource for therapeutic biomarker discovery in cancer cells. Nucleic Acids Res.

[41] Zhang X, Liao X, Wang M (2024). Inhibition of palmitoyltransferase ZDHHC12 sensitizes ovarian cancer cells to cisplatin through ROS-mediated mechanisms. Cancer Sci.

[42] Yuan M, Chen X, Sun Y (2020). ZDHHC12-mediated claudin-3 S-palmitoylation determines ovarian cancer progression. Acta Pharm Sin B.

[43] Tang F, Yang C, Li FP (2022). Palmitoyl transferases act as potential regulators of tumor-infiltrating immune cells and glioma progression. Mol Ther Nucleic Acids.

[44] Yu J, Cui X, Shao G (2026). LILRB3 inhibition reverses immunosuppression in glioma: a nanoparticle-based therapeutic strategy. J Nanobiotechnology.

[45] Wang L, Li M, Lian G (2024). Palmitoylation acts as a checkpoint for MAVS aggregation to promote antiviral innate immune responses. J Clin Invest.

[46] Wu S, Tang Y, Pan J (2025). ZDHHC5 as a central regulator in a palmitoylation-associated prognostic model for lung adenocarcinoma: insights from pan-cancer and experimental analyses. Front Immunol.

[47] Bao X, Zhang H, Wu W (2020). Analysis of the molecular nature associated with microsatellite status in colon cancer identifies clinical implications for immunotherapy. J Immunother Cancer.

